# Effects of Different Combinations of Concentric and Eccentric Resistance Training Programs on Traditional and Alternative Hamstrings-to-Quadriceps Ratios

**DOI:** 10.3390/sports7100221

**Published:** 2019-10-12

**Authors:** Cassio V. Ruas, Ronei S. Pinto, Guy G. Haff, Camila D. Lima, Lee E. Brown

**Affiliations:** 1 Centre for Exercise and Sports Science Research (CESSR), School of Medical and Health Sciences, Edith Cowan University, 270 Joondalup Dr, Joondalup 6027, Australia; g.haff@ecu.edu.au (G.G.H.); c.depauladelima@ecu.edu.au (C.D.L.); 2 Exercise Research Laboratory, School of Physical Education, Physioteraphy and Dance, Universidade Federal do Rio Grande do Sul, Rua Felizardo 750, Porto Alegre 90690-200, Brazil; ronei.pinto@ufrgs.br; 3 Center for Sport Performance and Human Performance Lab, Department of Kinesiology, California State University, Fullerton, 800 N State College Blvd, Fullerton, CA 92831, USA; leebrown@fullerton.edu

**Keywords:** muscle size, rate of torque development, muscle activation, peak torque, H:Q ratio, muscle imbalance, resistance training, injury prevention

## Abstract

Resistance training is often recommended for combined increases in traditional and alternative hamstrings-to-quadriceps (H:Q) ratios in order to reduce knee strength imbalance and associated hamstrings and knee ligament injury risk. The aim of this study was to investigate the effect of different concentric and eccentric resistance training programs on traditional and alternative H:Q ratios. Forty male volunteers were assigned to one of 4 groups: concentric quadriceps and concentric hamstrings (CON/CON, n = 10), eccentric quadriceps and eccentric hamstrings (ECC/ECC, n = 10), concentric quadriceps and eccentric hamstrings (CON/ECC, n = 10), or no training (control (CNTRL), n = 10). Traditional conventional (CR) and functional (FR), alternative rate of torque development (RTD), muscle size (MS), and muscle activation (MA) H:Q ratios were measured before and after six weeks of unilateral nondominant knee extension–flexion resistance training performed on an isokinetic dynamometer. The ECC/ECC training significantly increased FR (pre = 0.75 ± 0.11; post = 0.85 ± 0.15), whereas the lack of training (CNTRL) decreased the RTD H:Q ratio (pre = 1.10 ± 0.67; post = 0.73 ± 0.33). There were no differences between groups for the other traditional and alternative ratios following resistance training protocols. These findings suggest eccentric exercise for quadriceps and hamstrings as the most beneficial training program for inducing increases in the traditional FR. However, different resistance training strategies may be needed to also elicit increases in the alternative RTD, MS, and MA H:Q ratios for fully restoring muscle balance and reducing potential hamstrings and knee ligament injury risk.

## 1. Introduction

Anterior cruciate ligament (ACL) and hamstring strain injuries are common lower extremity noncontact injuries in sports. These may occur when the hamstrings fail to produce enough strength to decelerate high anterior tibial shear and rotation movements induced by maximal quadriceps strength actions [1,2,3,4]. Therefore, the assessment of the hamstrings-to-quadriceps (H:Q) muscle strength ratio calculated by peak torque (PT) is often used to screen imbalances between these muscle groups, which could be associated with injury risk [2,3,5]. A few studies have shown that athletes with low H:Q strength ratios are more likely to sustain hamstring injuries during competition [5,6]. Additionally, prescription of resistance training interventions focused on reducing H:Q muscle strength imbalance have been recommended to prevent noncontact injuries in sports [2,3,5] and to rehabilitate individuals with complete ACL tears [7]. However, since noncontact injuries are multifactorial, recent research has questioned if the H:Q ratio calculated by PT should be used as a single measurement of injury risk [8,9].

Traditional H:Q strength ratios can be classified as conventional or functional based upon how they are calculated. The conventional ratio is calculated by the balance between hamstring and quadriceps concentric (CON) PT, whereas the functional ratio calculates balance between hamstring eccentric (ECC) and quadriceps CON PT. The CR is commonly used to describe the absolute muscle strength relationship between hamstrings and quadriceps [1,3]. In contrast, the FR has been recommended as a more relevant and functional estimate of knee strength balance, as it considers the deceleration performed by hamstrings ECC torque during quadriceps CON muscle actions [2,4]. A CR or FR that is less than 0.6 and 1.0, respectively, have been suggested to identify potential risk of future hamstring strain and ACL injuries [1,4,9] for the general population. However, recent studies have questioned if standard cut-off/normative values and predictive models based on H:Q ratios are valid for different sporting populations [2,10].

Additionally, since these traditional methods of calculating H:Q ratios only account for knee balance based on the maximum strength capacity (i.e., PT), recent investigations have proposed alternative methods of determining H:Q ratios that consider balance between other neuromuscular variables that can influence the antagonist to agonist muscle relationship. This is the case of H:Q ratios calculated by rate of torque development (RTD) [11,12,13,14], muscle size (MS) [15,16,17], and muscle activation (MA) [18,19]. Indeed, a recent review has suggested that the use of these alternative H:Q ratios combined with the traditional H:Q ratios calculated by PT may provide a more comprehensive assessment of an individual’s H:Q muscle balance and associated injury risk [9].

Previous studies have suggested that future longitudinal investigations should be performed to provide greater insight about the most appropriated resistance training program for reducing potential injury risk as indicated by increased traditional and alternative H:Q ratios [9,14,16]. However, to our knowledge, no study has investigated the effects of different resistance training programs on both traditional and alternative H:Q ratios. Therefore, the aim of this study was to investigate the effects of different resistance training protocols involving CON and ECC muscle actions on traditional (CR and FR) and alternative (RTD, MS and MA) H:Q ratios. The results of this study may indicate the most advantageous resistance training intervention strategy for increases in both traditional and alternative H:Q ratios as measurements of muscle balance and associated injury prevention.

## 2. Materials and Methods

### 2.1. Participants

Forty healthy men (age: 22.9 ± 2.3 years, mass: 70.7 ± 11.0 kg, height: 174.3 ± 6.9 cm) volunteered for this study. Participants reported participating in sport and recreational activities, but they did not perform any endurance or resistance training programs for 3 months prior to initiation of the study. This ensured participants had similar low levels of strength before participating in the study. Therefore, based on the classification used in previous studies [20,21], participants may be considered as being recreationally or physically active. Participants reported having no knee injuries and were asked to refrain from exercise 2 days before starting the study. All participants completed a pre-exercise medical questionnaire to identify any injury or illness that could influence testing and performance, and read and signed a University Institutional Review Board-approved (HSR-14-0454) informed consent form based on the Declaration of Helsinki.

### 2.2. Experimental Design

**Figure 1:**
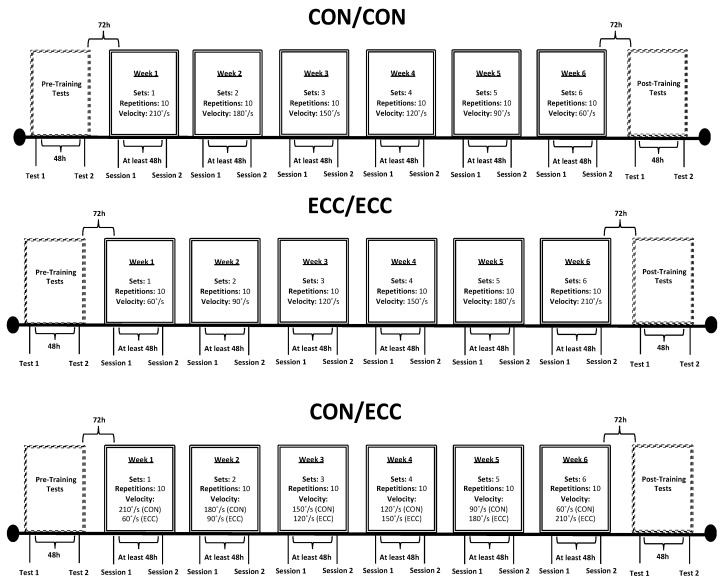
Study design and training programs for each group, including training sets, repetitions, and velocity per week for concentric (CON) and eccentric (ECC) muscle actions, and time intervals between pre- and post-testing and training sessions.

Participants were randomly allocated to one of four groups (n = 10 participants per group): CON quadriceps and CON hamstring (CON/CON), ECC quadriceps and ECC hamstring (ECC/ECC), CON quadriceps and ECC hamstring (CON/ECC), or no training (control (CNTRL)). The three training groups performed six weeks of unilateral nondominant knee extension–flexion exercise on a Biodex isokinetic dynamometer (Figure 1). In this study, only the nondominant leg was trained and tested. Although the nondominant leg has a common weight-bearing role in sports to support actions of the dominant leg, which may lead to high incidence of knee ligament injuries [2,22,23], the H:Q muscle balance and associated injury risk on this leg are rarely investigated. Therefore, the nondominant leg CON and ECC quadriceps and hamstrings PT, RTD, MS, and MA were measured before and after training for all groups. Traditional (CR and FR) and alternative (RTD, MS, and MA) H:Q ratios were then calculated.

### 2.3. Pretesting

All pretesting sessions were performed on two separate occasions (separated by 48 h). On day one, participants were tested for quadriceps and hamstrings muscle thickness (MT), followed by isometric maximal testing, which included RTD and MA measurements. On day two, participants performed isokinetic quadriceps and hamstrings CON and ECC maximal testing for PT measurements. The training sessions started 72 h after the last pretesting.

#### 2.3.1. Ultrasound Measurements

Participants laid supine on a table with legs relaxed for 10 min in order to allow body fluids to stabilize. An experienced researcher measured MT images of quadriceps rectus femoris (RF), vastus intermedius (VI), vastus lateralis (VL), and vastus medialis (VM) and hamstrings biceps femoris long head (BF_lh_), semitendinosus (ST), and semimembranosus (SM) muscles of the nondominant leg using a real-time portable B-mode ultrasound device (GE LOGIQ^TM^ e, GE Healthcare, Chicago, IL, USA). For this, a linear array probe (code 12L-RS, variable frequency band 4.2–13.0 MHz, GE Healthcare, Chicago, IL, USA) was placed perpendicular to quadriceps and hamstrings muscles at 50% of the distance between the greater trochanter and the lateral condyle of the femur, except for the VM, which was at 30% of this same distance [24,25]. The settings for gain, depth, and frequency (52 dB, 9 cm, 12 MHz) were maintained for all images for pre- to post-testing. For most muscles, MT measurements were performed by the widest distance between the adipose muscle upper and lower fascia. However, for the VI, it was measured as the widest distance between the bone and the muscle upper interface [24,25,26]. Three MT measurements were performed for each muscle, and the average was calculated. All analyses were performed by using ImageJ software (Version 1.48v, National Institutes of Health, Bethesda, Rockville, MD, USA). Interday reliability for MT was tested from the first ten participants of the study, who attended the lab one day before the pretesting intervention. Intraclass correlation coefficients (ICCs) for quadriceps and hamstrings MTs ranged from 0.97–0.99, which are considered as very high reliability [27]. Additionally, the three pre- and post-testing MT measurements for quadriceps and hamstrings muscles had maximal coefficients of variation (CV) of 0.33% and 0.23% (RF), 0.45% and 1.27% (VI), 0.78% and 0.98% (VL), 0.29% and 0.01% (VM), 0.25% and 0.36% (BF_lh_), 0.69% and 0.48% (ST), and 0.52% and 0.44% (SM). Previous studies have determined muscle thickness (MT) as a measure of muscle size (MS) [26,28]. Therefore, hamstrings MS was considered as the sum of BF_lh_ + ST + SM MT, and quadriceps MS was considered as the sum of RF + VI + VL + VM MT [29]. These values were used for further analyses.

#### 2.3.2. Isometric Maximal Testing, MA, and RTD

Maximal quadriceps and hamstrings isometric strength for the nondominant leg was measured on a Biodex System 2 isokinetic dynamometer (Biodex Medical Systems, Shirley, NY, USA). Participants sat comfortably on the seat of the machine, having straps across chest, thighs, and hips. The lateral femoral condyle of the test leg was aligned to the machine’s axis of rotation. The ankle was attached to the lever arm by an ankle cuff positioned slightly above the medial malleolus [26,30,31]. Prior to testing, participants performed a specific warm-up consisting of 10 repetitions of isokinetic knee extension–flexion CON muscle actions at 180°/s through 90° of range of motion (0° = full extension). Additionally, three submaximal preliminary isometric repetitions were completed before each test [26,30]. Testing consisted of three repetitions of 3 s maximal isometric quadriceps strength followed by three repetitions of 3 s maximal hamstrings strength, respectively, separated by 5 min rest. Participants were instructed to push and pull as hard as possible for each muscle action. The average of 3 repetitions for each muscle action was used for further analyses. The three pre- and post-testing isometric PT measurements had maximal CVs of 1.84% and 0.29% (quadriceps) and 1.66% and 2.86% (hamstrings). The RTD was calculated as isometric PT/time to reach isometric PT [31]. The three pre- and post-testing RTD measurements had maximal CVs of 10.41% and 15.28% (quadriceps) and 17.61% and 4.40% (hamstrings).

During the test, participants were fitted with 2 bipolar (3.5 cm center-to-center) disposable electromyography (EMG) surface electrodes (EL500 silver-silver chloride; BIOPAC Systems, Inc., Goleta, CA, USA) on their quadriceps VL and hamstrings BF_lh_ muscles for MA measurements. The same anatomical sites used for ultrasound measurements were used for placement of electrodes. The areas surrounding these sites were shaved, abraded, and cleaned, and electrodes were replaced if high levels of noise were identified, which may indicate high skin impedance. Raw EMG signals were filtered (10–500 Hz; fourth-order Butterworth) and amplified (bandwidth = 1–500 Hz amplifier) using a Myopac EMG device (MPRD-101; Run Technologies, Mission Viejo, CA, USA) with a sampling rate of 1 kHz. The calculation of the root mean square (RMS) was performed over a 1 s plateau (i.e., time interval starting as soon as maximal plateau was achieved) of each knee flexion and extension maximal isometric torque by using a custom LabVIEW (V. 2014, National Instrument Corporation, Austin, TX, USA). The average of 3 EMG values for each muscle action was considered for further analysis. The three pre- and post-testing EMG measurements had maximal CVs of 6.02% and 1.07% (quadriceps) and 1.69% and 6.26% (hamstrings).

#### 2.3.3. Isokinetic Maximal Testing

Participants were tested for maximal quadriceps and hamstrings CON and ECC muscle actions for the nondominant leg on the same Biodex isokinetic dynamometer and using the same procedures as the isometric maximal test. Before testing, participants performed a specific isokinetic warm-up consisting of 10 CON knee extension–flexion repetitions at 180°/s, followed by three repetitions at 60°/s through 90° of range of motion (0° = full extension). Testing consisted of 5 maximal CON and 5 ECC knee extension–flexion repetitions at 60°/s, separated by 2 min rest. Isokinetic torque data were collected using LabVIEW software (sampling rate: 1 kHz). The highest quadriceps and hamstrings CON and ECC PT values across all repetitions were considered for further analysis. 

#### 2.3.4. Traditional and Alternative H:Q Ratios

Traditional H:Q ratios were represented by CR and FR. CR was calculated by dividing hamstrings CON PT by quadriceps CON PT, whereas FR was calculated by dividing hamstrings ECC PT by quadriceps CON PT. Alternative ratios were represented by RTD, MS, and MA H:Q ratios. RTD H:Q ratios were calculated by dividing hamstrings RTD by quadriceps RTD values, MS H:Q ratios were calculated by dividing hamstrings MS by quadriceps MS values, and MA H:Q ratios were calculated by dividing hamstrings (BF_lh_) EMG by quadriceps (VL) EMG values.

### 2.4. Training Sessions 

The training sessions were performed for 6 weeks (2 sessions a week separated by at least 48 h). The first training session began 72 h after the second day of pretesting. Participants sat on the same Biodex used for isokinetic testing, using the same set-up and warm-up procedures. The CON/CON group started the first week of training by performing 1 set of 10 maximal repetitions at 210°/s for quadriceps and hamstrings. The ECC/ECC group started the first week of training by performing 1 set of 10 maximal repetitions at 60°/s for quadriceps and hamstrings. The CON/ECC group started the first week of training by performing 1 set of 10 maximal CON repetitions at 210°/s for quadriceps and 1 set of 10 maximal ECC repetitions at 60°/s for hamstrings. The training intensity was increased every week by increasing the isokinetic angular velocity for eccentric and decreasing it for concentric in 30°/s increments [26,31,32]. Additionally, training volume was increased by adding 1 set every week [26,31]. CON and ECC resistance exercises for all groups were performed with the use of an isokinetic dynamometer, which allows maximal torque production throughout a predetermined range of motion. Participants were instructed and verbally encouraged to always perform with maximal effort across CON and/or ECC muscle action repetitions for every training session through 90° of range of motion (0° = full extension), regardless of the training program performed. Therefore, the maximal force applied against the machine resulted in an equal reaction force through accommodating resistance within the selected fixed isokinetic speed performed every week [33]. The training program designs are presented in Figure 1. The CNTRL group did not perform any resistance training program, but they returned to the lab for testing after six weeks.

### 2.5. Post-Testing

Post-testing sessions were performed on two days (separated by 48 h) with the same order and procedures as the pretesting sessions. All tests were performed 72 h after the last training session.

### 2.6. Statistical Analyses

Normality of data was verified by the Shapiro–Wilk test. A 2 × 2 × 2 × 4 (muscle × action × time × group) mixed factor ANOVA was used to compare PT. Three 2 × 2 × 4 (muscle × time × group) mixed factor ANOVAs were used to compare RTD, MS, and MA. A 5 × 2 × 4 (ratio × time × group) mixed factor ANOVA was used to compare all traditional (CR and FR) and alternative (RTD, MS, and MA) H:Q ratios. Significant interactions and main effects were followed up by paired *t* tests and posthoc analysis (Fisher’s least significant difference), respectively. Data are expressed as mean and SD. An a priori alpha level of 0.05 determined statistical significance. Analyses were performed with SPSS 21.0 (Statistical Package for Social Sciences, Chicago, IL, USA). Effect sizes were calculated by Cohens *d.* According to Rhea [34], values <0.50 are considered trivial, 0.50–1.25 small, 1.25–1.9 moderate, and >2.0 large for untrained participants.

## 3. Results

Results and effect sizes of PT, RTD, MT, and MA are presented in Table 1.

For PT, there was a significant three-way interaction (muscle × time × group). This was followed up with four two-way ANOVAs (muscle × time), one for each group. All groups demonstrated significant interactions, which were followed up with two paired *t* tests per group. For groups CON/CON and CNTRL, hamstrings PT increased from pre- to post-testing (p < 0.05), but quadriceps PT was not different from pre- to post-testing (p > 0.05). For groups ECC/ECC and CON/ECC, hamstrings and quadriceps PT increased from pre- to post-testing (p < 0.05).

For RTD, there were no interactions. However, there was a main effect for muscle (p < 0.001), where quadriceps was greater than hamstrings.

For MS, there was a significant two-way interaction (time × group). This was followed up with one paired *t* test per group. For groups CON/CON, ECC/ECC, and CON/ECC, MS increased from pre- to post-testing (p < 0.05). For group CNTRL, MS decreased from pre to post-testing (p < 0.05).

For MA, there was a significant two-way interaction (time × group). This was followed up with one paired t-test per group. For group CON/CON, MA increased from pre to post-testing (p < 0.05). For groups ECC/ECC, CON/ECC, and CNTRL, MA did not differ from pre- to post-testing (p > 0.05).

**Figure 2:**
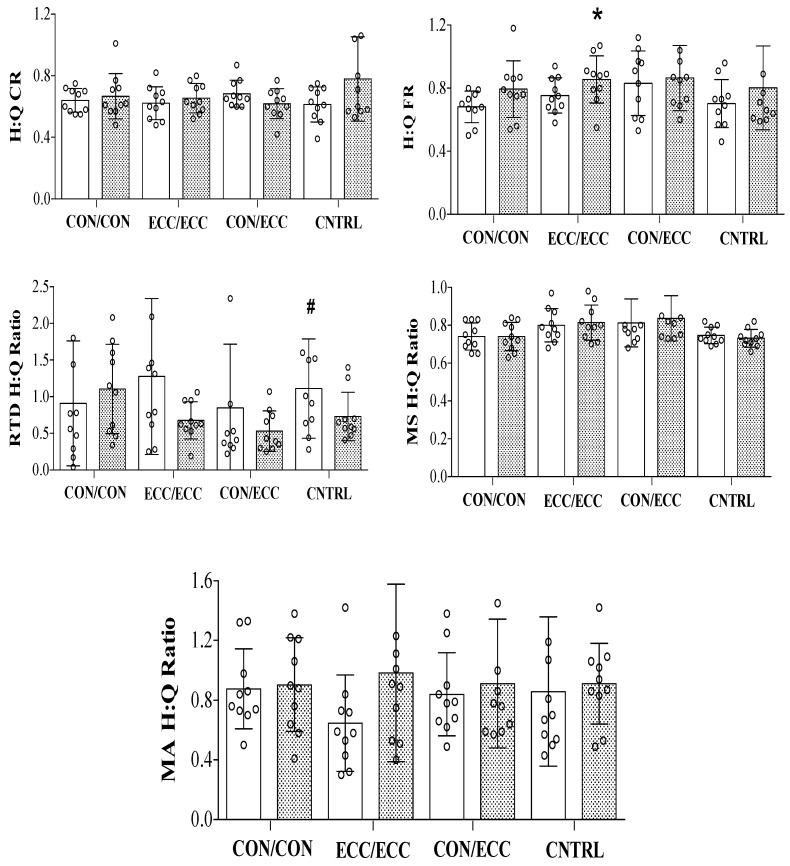
Means and SDs of hamstrings-to-quadriceps (H:Q) conventional (CR), functional (FR), rate of torque development (RTD), muscle size (MS), and muscle activation (MA) ratios between pre- and post-tests for concentric/concentric (CON/CON), eccentric/eccentric (ECC/ECC), concentric/eccentric (CON/ECC), and control (CNTRL) groups. The open circles demonstrate individual pre- to post-testing data.* Significantly greater than pre-test. # Significantly greater than post-test.

For H:Q ratios, there was a significant three-way interaction (ratio × time × group). This was followed up with four two-way ANOVAs (ratio × time), one for each group. For groups CON/CON and CON/ECC, there were no interactions or main effects. Groups ECC/ECC and CNTRL demonstrated interactions, which were followed up with five paired *t* tests per group. For group ECC/ECC, FR increased from pre- to post-testing (p < 0.05), but there were no differences for the other ratios (p > 0.05). For CNTRL, RTD H:Q ratios decreased from pre- to post-testing (p < 0.05), but there were no differences for the other ratios (p > 0.05) (Figure 2).

## 4. Discussion

The aim of the present study was to investigate the effects of different resistance training protocols involving CON and ECC muscle actions on traditional and alternative H:Q ratios. Results revealed that ECC/ECC training led to increases in FR, but no other resistance training protocols involving CON and ECC muscle actions were effective at increasing traditional or alternative H:Q ratios. These findings suggest ECC exercise may be the most beneficial training intervention impacting the quadriceps and hamstrings and inducing increases in the traditional FR. However, given the importance of increases in both traditional and alternative H:Q ratios for fully restoring muscle balance and reducing potential hamstrings and knee ligament injury risk, different strategies of resistance training may be needed in order to induce increases in the alternative RTD, MS, and MA H:Q ratios.

Previous studies have recommended that resistance training interventions should focus on increasing hamstrings ECC strength in order to increase H:Q muscle strength balance [2,4,5,31]. This contention is based on the main deceleration role of the knee flexors during anterior tibial shear and rotation movements of the knee extensors [1,2,3,4]. The hamstrings also have a major role in stabilizing knee and hip joints during sporting actions [35]. Since hamstring strains and ACL injuries may be a result of low ECC force output, hamstrings ECC resistance training has been suggested as the most effective in reducing injury risk [36,37]. For instance, Croiser et al. [5,37] in two longitudinal studies found that professional soccer players that had manual, isotonic, and/or isokinetic resistance training focused on normalizing H:Q muscle strength imbalances during preseason, with particular emphasis on hamstrings eccentric muscle actions, reduced their hamstrings injury rate in the subsequent 9–12 months of playing season. Similarly, Asking et al. [35] investigated the effect of 10 weeks of resistance training with additional eccentric overload exercises for hamstrings (performed on a flywheel ergometer) on strength, functionality, and injury incidence in professional soccer players. Players that performed the additional training program had ~15% greater hamstrings eccentric peak torque, ~2.4% lower flying 30 min speed time, and ~47% lower hamstrings injury incidence in the following 10 months of the playing season compared to counterparts that did not receive additional eccentric resistance training. Li et al. [7] also found that recreational athletes with arthroscopically confirmed ACL tears increased their functional ability by ~17.3% following 6 weeks of resistance training on an isokinetic dynamometer targeted at normalizing H:Q ratios via hamstrings strengthening. Previous studies have also demonstrated that ECC training for hamstrings only [36] or hamstrings and quadriceps [31] is effective at increasing the FR. This usually results from increases in hamstrings ECC strength without concomitant quadriceps CON strength increases [31,36].

Our results are in agreement with most of these studies Although all training groups had significant hamstrings and/or quadriceps torque increases, the achieved ES revealed that the ECC/ECC training was the only intervention that exhibited a moderate magnitude of change for hamstrings ECC PT. The other groups presented only small, trivial magnitudes for quadriceps and hamstrings CON and ECC PT. Therefore, the moderate hamstrings ECC PT to small quadriceps CON PT magnitude increases may explain the significant increases in FR found for the ECC/ECC group. This may also help explain the lack of changes in the traditional but less functionally relevant CR. Additionally, it is also possible that quadriceps ECC training reduces hamstrings co-contraction [38]. This may explain why the ECC/ECC group showed greater improvements in FR than the CON/ECC group. Therefore, ECC training for hamstrings and quadriceps may be of paramount importance for stimulating increases in H:Q muscle balance and injury prevention. Since we only tested the nondominant leg, our results may be important for athletes from sports that require weight-bearing of the nondominant leg to support actions of the dominant leg, which may lead to greater risk of knee ligament injuries [2,22]. However, recent evidence has indicated that H:Q ratios calculated by PT alone may be weak indicators of future injury incidence in sports [8,39], and that this relationship may also be influenced by other neuromuscular variables, such as RTD, MS, MA, muscle fatigue, and torque produced at multiple angles of ROM [9,11,13,14,15,16,17,21,40,41]. Therefore, because of the multifactorial nature of hamstring strain ACL tears [8,42], increasing traditional and alternative H:Q ratios (based on more than one of these neuromuscular variables) have been proposed as potential strategies for enhancing performance and reducing injury risk [9]. 

MS increases have been thought to be a primary determinant of strength gains following resistance training [43]. Because of this relationship, a few studies have suggested that a reduced MS H:Q ratio can also indicate an increased knee strength imbalance and potential risk of injury [15,16,17]. For instance, Evangelidis et al. [15] demonstrated that quadriceps MS explained about 30% to 31% of CON PT, and that hamstrings MS explained about 48% to 58% of ECC PT. This resulted in the H:Q MS ratio as having a positive association and explained about 12% to 31% of the FR. Therefore, the measurement of this ratio via imaging techniques has been highlighted as an important alternative method for identifying H:Q muscle imbalance [9], and resistance training interventions that are focused on increasing hamstrings eccentric strength and size have been recommended for an increased H:Q muscle balance and reduction in knee injury risk [15,16,17]. Our results demonstrated that none of the tested resistance training strategies involving CON or ECC muscle actions was able to modify the MS H:Q ratio. This may have occurred because all of the resistance training interventions resulted in similar increases in both quadriceps and hamstrings MS. Additionally, test–retest reliability and CV values demonstrated that MT was highly reliable between days and measures. Therefore, the decreases in MS we found for the CNTRL group could be most likely related to the lack of training than any variation of the measurements. We were unable to compare our results with previous research because of the lack of longitudinal studies investigating this ratio after resistance training. However, our results may suggest that a greater volume of training may be necessary for hamstrings to elicit greater H:Q MS balance.

Hamstrings strains and ACL injuries have been thought to occur in the early phases of sporting movements. Therefore, the RTD H:Q ratios have been proposed as an alternative method to calculate the knee strength balance necessary during explosive sporting movements [11,12,13]. Interestingly, several studies have found that H:Q ratios calculated by PT and RTD are significantly different and not correlated [11,12,13]. For instance, Greco et al. [11] found that there was no significant correlation between PT and RTD H:Q ratios (at 0–50 ms) in professional soccer players. However, soccer players with high torque levels had about 20% to 23% greater CR and RTD H:Q ratios compared to soccer players with low torque levels. They concluded that, although there may be similarities between the mechanisms underpinning explosive and maximal strength, these ratios should be analyzed separately for clinical use. Similarly, Hannah et al. [12] found that the RTD H:Q ratio at 50 ms was ~56% lower than H:Q ratio calculated by isometric PT. This was explained because the hamstrings electromechanical delay was twice as long as that of the quadriceps (44.0 vs. 22.6 ms), which could lead to knee joint instability in the early phase of contraction and increased ACL injury risk. Grazioli et al. [13] also found that soccer players had ~16% lower early RTD H:Q ratio (50 ms) after playing a soccer match, but their late RTD (200 ms) and PT H:Q ratios remained unchanged. Additionally, RTD and PT H:Q ratios were not correlated before or after match-induced fatigue. Although we did not measure RTD at 50 ms, our results are partially in agreement with these findings, as increases in FR were found following ECC/ECC, but none of our resistance training protocols increased H:Q ratios calculated by RTD. Therefore, based on the consistent body of research showing distinct clinical and functional relevance between these ratios calculated by PT and RTD [9,11], a different resistance training strategy (i.e., exploring hamstrings explosive strength) may be needed for increases in the RTD H:Q ratio. 

The MA H:Q ratio has been proposed as a method to quantify the hamstrings relative to quadriceps MA, which functionally occurs to decelerate the high anterior tibial shear and rotation movements induced by maximal knee extension movements [9,18,19]. An increased H:Q MA balance may indicate increased knee joint stability and reduced ligament injury risk [9]. To our knowledge, only two previous studies [18,19] have investigated MA H:Q ratio and found that hamstrings MA increased to approximately 30–35% of quadriceps MA towards maximal knee extension. The authors of these studies agreed that this may be a mechanism to increase knee joint stability during knee extension motion, improving movement efficiency [19] as well as protecting the ACL ligament [18]. Different from these studies, we only measured H:Q MA at the quadriceps VL and hamstrings BF_lh_ muscles during isometric muscle actions before and after different resistance training protocols, which may only describe an absolute relationship between hamstrings and quadriceps MA. Interestingly, agonist resistance training has been suggested to be effective at inducing neural changes, such as reductions in antagonist MA, to optimize force production [9,44]. However, although we found that the CON/CON training led to significant, similar increases between hamstrings and quadriceps MA, not affecting the MA H:Q ratio, the other interventions were not effective at increasing isometric agonist or antagonist MA. In agreement with our results, previous studies have shown that short-term resistance training (i.e., six to seven weeks) may not be sufficient to modify the antagonist–agonist MA mechanism [26,45]. Additionally, in a review study, Gabriel et al. [44] reported that modifications in antagonist MA following resistance training interventions are not fully understood, as the central nervous system needs to optimize for both joint integrity (increase in antagonist MA) and force production (decrease in antagonist MA). However, given the effectiveness of the ECC/ECC at increasing the FR, it is possible that this type of training may have modified MA H:Q ratio measured during dynamic contractions. Therefore, future studies investigating short- and long-term antagonist–agonist MA changes specific to the muscle action trained are needed to further test the effects of different resistance training protocols on modifying the MA H:Q ratio.

In this study, participants had preliminary repetitions before isometric (including RTD) and isokinetic maximal testing and received proper instruction and verbal encouragement to perform each test. However, it is possible that additional familiarization sessions performed before the intervention could have minimized the greater variability we found in some functional pretesting compared to post-testing measures between groups.

## 5. Conclusions

Our findings suggest that ECC training for quadriceps and hamstrings is an advantageous resistance training program for inducing increases in the traditional (FR) H:Q ratio, which could be effective for rehabilitation and injury prevention purposes. However, given the importance of increases in both traditional and alternative H:Q ratios for fully restoring muscle balance and reducing potential hamstrings and knee ligament injury risk, different strategies of resistance training may be needed for also inducing increases in the alternative RTD, MS, and MA H:Q ratios. 

## Figures and Tables

**Table 1:** Means and SDs of quadriceps concentric peak torque (Q CON PT), quadriceps eccentric peak torque (Q ECC PT), hamstrings concentric peak torque (H CON PT), hamstrings eccentric peak torque (H ECC PT), quadriceps rate of torque development (Q RTD), hamstrings rate of torque development (H RTD), quadriceps muscle activation (Q MA), and hamstrings muscle activation (H MA) between pre- and post-tests for concentric/concentric (CON/CON), eccentric/eccentric (ECC/ECC), concentric/eccentric (CON/ECC), and control (CNTRL) groups. Data collapsed across action (i.e., muscle × time interaction) and muscle (time × group interaction) are presented. Effect sizes are presented for pre- and post-test comparisons.

	CON/CON			ECC/ECC			CON/ECC			CNTRL		
	Pre	Post	ES	Pre	Post	ES	Pre	Post	ES	Pre	Post	ES
Q CON PT	226.23 ± 47.05	231.84 ± 51.77	0.12	206.44 ± 46.84	240.44 ± 60.13	0.73	197.51 ± 38.28	222.06 ± 43.81	0.64	200.32 ± 39.00	202.39 ± 35.48	0.05
Q ECC PT	229.14 ± 63.15	306.83 ± 49.05	1.23	235.96 ± 64.90	312.67 ± 52.64	1.18	243.13 ± 50.07	279.90 ± 72.32	0.61	230.67 ± 58.86	223.62 ± 79.69	−0.12
Q PT (Collapsed, Action)	340.80 ± 70.19	385.26 ± 63.77	0.63	324.42 ± 77.16	396.77 ± 81.95 *	0.94	319.08 ± 63.59	362.01 ± 75.90 *	0.68	315.66 ± 60.35	314.20 ± 62.32	−0.024
H CON PT	143.79 ± 29.04	149.41 ± 23.84	0.19	127.65 ± 31.69	154.21 ± 26,68	0.84	135.17 ± 20.18	143.52 ± 21.22	0.41	124.91 ± 36.04	155.57 ± 61.81	0.85
H ECC PT	152.39 ± 31.31	179.86 ± 43.46	0.88	153.88 ± 31.21	198.31 ± 17.44	1.42	160.28 ± 35.98	186.67 ± 33.40	0.73	140.17 ± 37.29	158.55 ± 45.01	0.49
H PT (Collapsed, Action)	219.98 ± 42.89	396.76 ± 62.41 *	4.12	204.58 ± 46.19	411.83 ± 57.60 *	4.49	215.31 ± 35.43	373.23 ± 82.80 *	4.46	195.99 ± 55.37	302.90 ± 88.76 *	2.02
Q RTD	196.73 ± 144.91	162.53 ± 132.73	−0.24	142.85 ± 149,77	225.91 ± 130.02	0.55	259.33 ± 128.33	304.99 ± 107.39	0.36	163.21 ± 67.40	222.85 ± 134.65	0.88
H RTD	102.99 ± 53.70	135.18 ± 49,53	0.60	111.84 ± 71.51	149.69 ± 88.82	0.53	157.57 ± 99.52	168.39 ± 101.24	0.11	170.00 ± 124.29	146.16 ± 78.80	−0.19
Q MT	140.86 ± 9.80	149.31 ± 14.96	0.86	135.72 ± 10.84	154.47 ± 8.22	1.73	130.52 ± 14.96	138.60 ± 14.76	0.54	135.25 ± 7.51	131.55 ± 9.93	−0.49
H MT	104.20 ± 8.77	110,48 ± 15.19	0.72	108.84 ± 16.15	125.62 ± 15.90	1.04	104.71 ± 12.59	115.39 ± 14.10	0.85	100.88 ± 7.96	96.32 ± 9.80	−0.57
MT (Collapsed, Muscle)	192.86 ± 11.73	204.56 ± 20.78 *	0.99	190.14 ± 17.42	217.28 ± 13.73 *	1.56	182.88 ± 17.45	196.29 ± 18.33 *	0.77	185.69 ± 10.61 ^#^	179.72 ± 14.22	−0.56
Q MA	0.49 ± 0.22	0.57 ± 0.21	0.35	0.56 ± 0.19	0.45 ± 0.22	−0.60	0.52 ± 0.26	0.41 ± 0.13	−0.41	0.45 ± 0.20	0.45 ± 0.17	0.033
H MA	0.40 ± 0.15	0.51 ± 0.25	0.72	0.37 ± 0.26	0.37 ± 0.21	0.0097	0.44 ± 0.27	0.40 ± 0.28	0.00075	0.33 ± 0.14	0.39 ± 0.14	0.39
MA (Collapsed, Muscle)	0.65 ± 0.24	0.82 ± 0.30 *	0.73	0.65 ± 0.32	0.64 ± 0.31	−0.04	0.70 ± 0.38	0.63 ± 0.24	−0.18	0.56 ± 0.20	0.65 ± 0.22	0.45

* Significantly greater than pretest. # Significantly greater than post-test.
